# Supplementary material for: Mesoscopic calcium imaging in a head-unrestrained male non-human primate using a lensless microscope
Source: Nat Commun. 2024 Feb 10;15:1271. doi: 10.1038/s41467-024-45417-6 (PMC10858944; doi:10.1038/s41467-024-45417-6)
Supplement: Supplementary file 1 — Supplementary Information [file 41467_2024_45417_MOESM1_ESM.pdf]

Contents

Supplementary figures

Supplementary Fig. 1 | Weights of reported head-mounted devices on different animal models.

Supplementary Fig. 2 | FOV comparison with current miniaturized fluorescence microscopes used for imaging head-unrestrained NHPs.

Supplementary Fig. 3 | Phase mask design and fabrication.

Supplementary Fig. 4 | Resolution characterization over the whole FOV.

Supplementary Fig. 5 | In vivo imaging setups and epifluorescence images.

Supplementary Fig. 6 | Calibration of spatially variant PSFs.

Supplementary Fig. 7 | Bio-FlatScopeNHP housing design and illumination profile.

Supplementary Fig. 8 | Comparison of different reconstruction models.

Supplementary Fig. 9 | Root-mean-square (RMS) maps for selecting ROIs.

Supplementary Fig. 10 | Additional results for spatial response profiles measured from V1 in the right hemisphere of a head-fixed NHP.

Supplementary Fig. 11 | Additional results for columnar-scale signals measured from V1 in the right hemisphere of a head-fixed NHP.

Supplementary Fig. 12 | Comparison between head-fixed and head-unrestrained imaging using Bio-FlatScopeNHP in the same imaging session.

Supplementary Fig. 13 | Stability of Bio-FlatScopeNHP imaging on a head-unrestrained macaque.

Supplementary Fig. 14 | Correlation coefficient between random maps and ground truth.

Supplementary Fig. 15 | Heartbeat artifact removal.

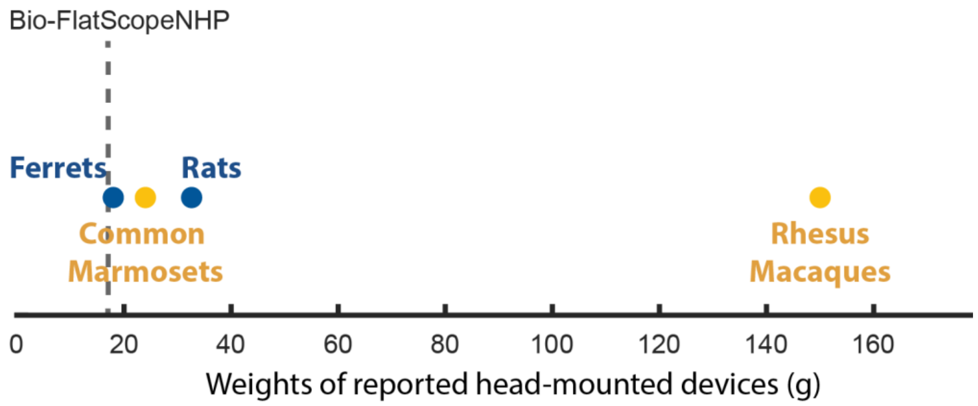

Supplementary Fig. 1 | Weights of reported head-mounted devices on different animal models. Bio-FlatScopeNHP has a lighter weight compared to the reported devices used on ferrets[1], rats[2], and NHPs including common marmosets[3] and rhesus macaques[4].

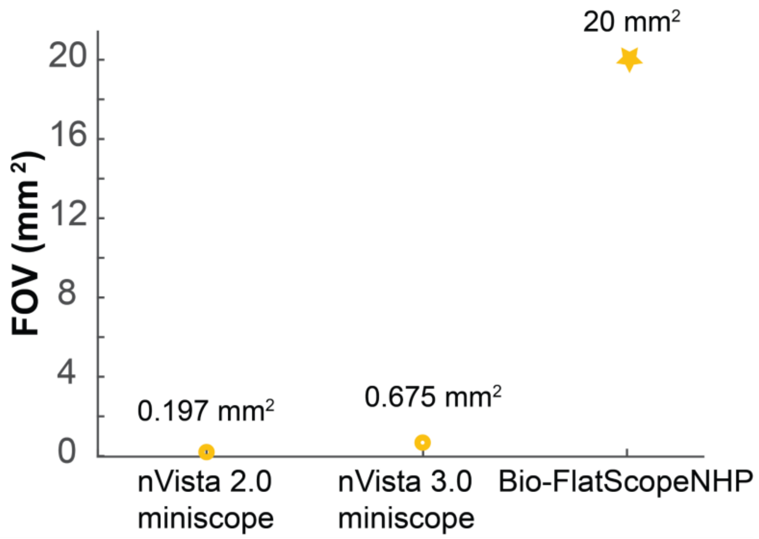

Supplementary Fig. 2 | FOV comparison with current miniaturized fluorescence microscopes used for imaging head-unrestrained NHPs (nVista 2.0 miniscope[5], nVista 3.0 miniscope[6]).

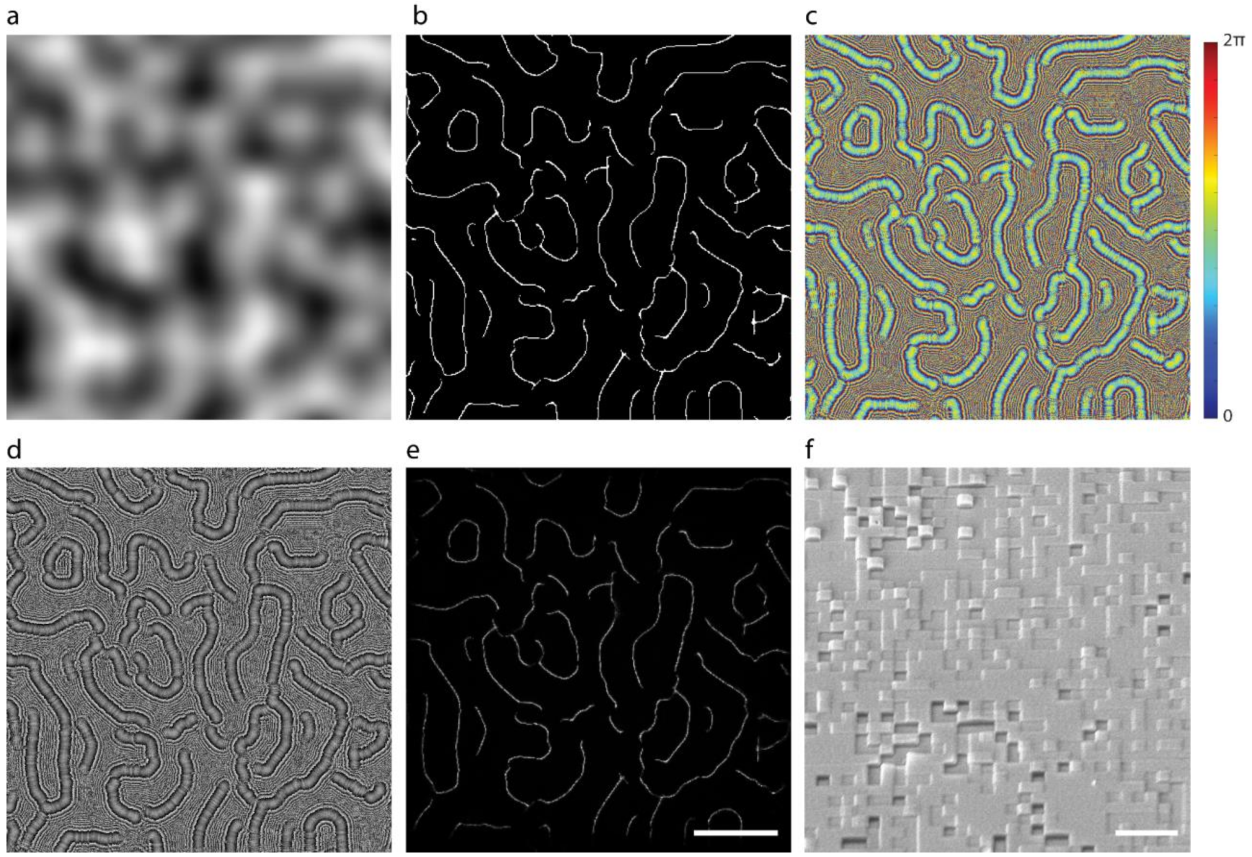

Supplementary Fig. 3 | Phase mask design and fabrication. a, Perlin noise pattern generated for PSF design. **b**, Designed PSF pattern by applying Canny edge detection to Perlin noise pattern. **c**, Phase map generated by the phase retrieval algorithm for the mask in order to produce the designed PSF pattern on the sensor. **d**, Height map generated with designed focal length for fabrication. **e**, Captured PSF at designed focal length. Scale bar, 500 μm. **f**, Zoomed scanned electron micrograph of a fabricated phase mask with 1 μm fabrication pixel size. Scale bar, 5 μm.

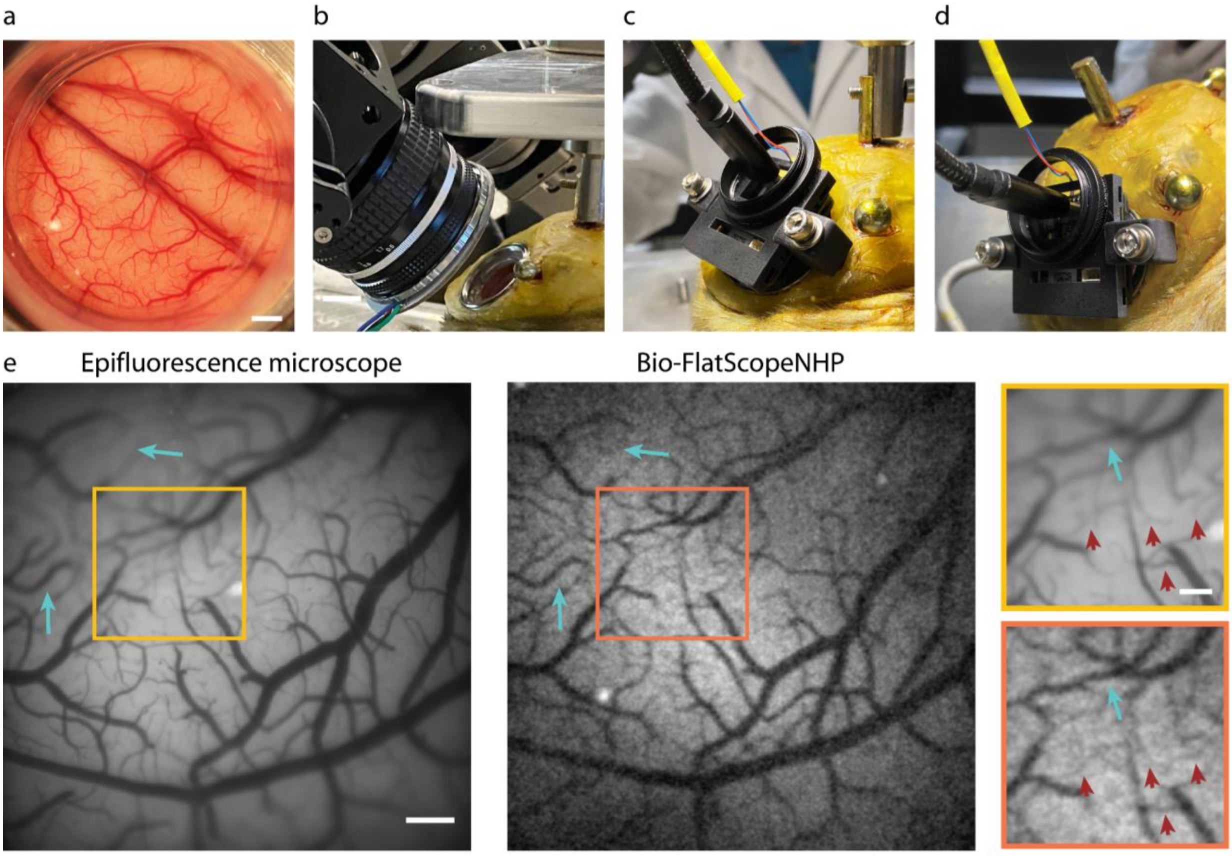

Supplementary Fig. 4 | In vivo imaging setups and epifluorescence images. a, Photo of one cranial window over V1 of a macaque monkey. Scale bar, 2 mm. **b**, Photo of in vivo imaging of a head-fixed macaque using the table-top widefield microscope. **c**, Photo of in vivo imaging of a head-fixed macaque using Bio-FlatScopeNHP. **d**, Photo of in vivo imaging of a head-unrestrained macaque using Bio-FlatScopeNHP. **e**, High resolution images of a head-fixed macaque captured in vivo by the ground truth widefield microscope and Bio-FlatScopeNHP. Scale bar, 500 μm. Blue arrows indicate regions in the table-top widefield microscope image that are out of focus, while these same areas remain sharply focused in our system. Zoom-in shows Bio-FlatScopeNHP can resolve similar small features as ground truth. Scale bar, 100 μm. Red arrows indicate small blood vessels with 10 μm - 20 μm diameters.

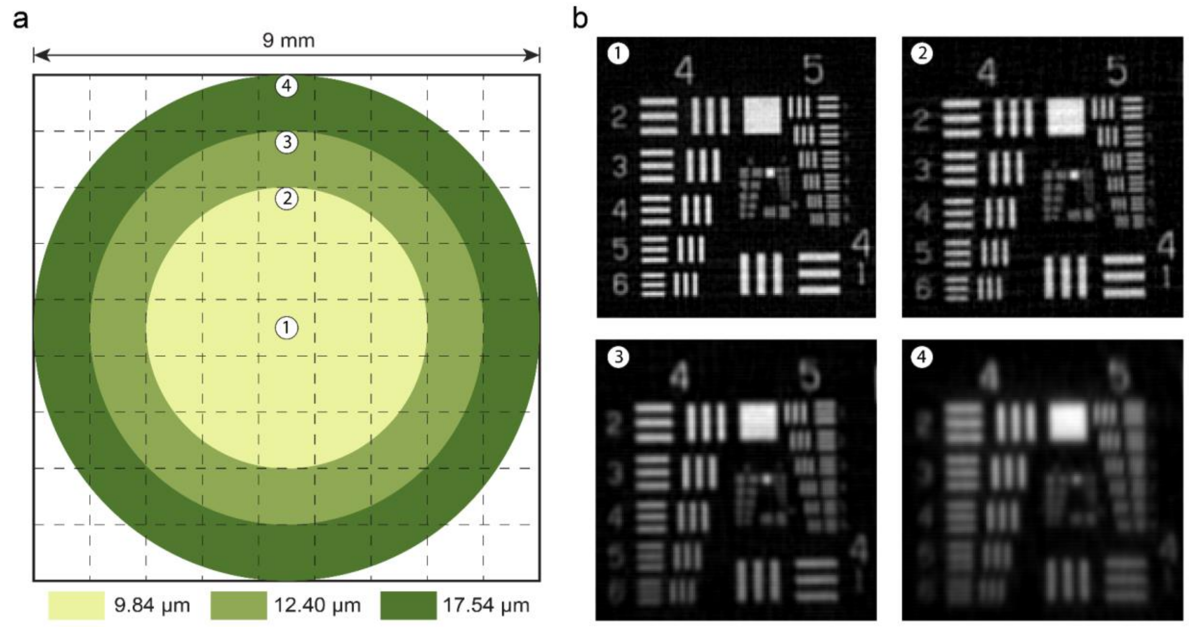

Supplementary Fig. 5 | Resolution characterization over the whole FOV. a, Resolution obtained within each specified area across the whole FOV. b, Resolution test target reconstructions using captures from the spatial position indicated in panel a.

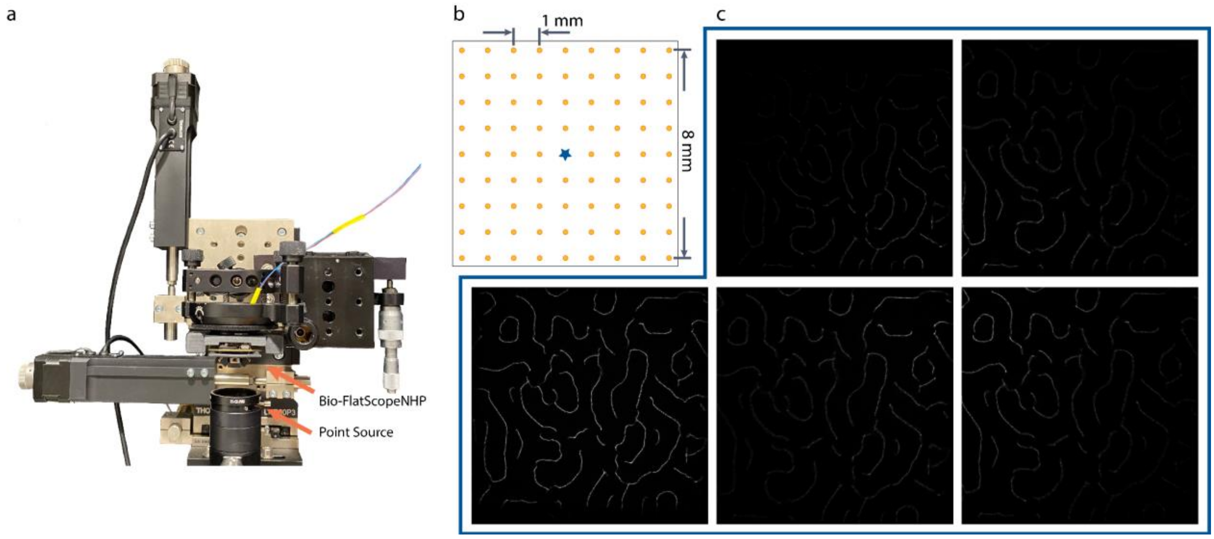

Supplementary Fig. 6 | Calibration of spatially variant PSFs. a, Photo of calibration setup for automatic calibration at xyz directions. **b**, Calibration positions (yellow dots) on a single depth. Blue star indicates the calibration position of center PSF used for fast reconstruction. **c**, Example PSFs captured at different positions at the 3 mm working distance.

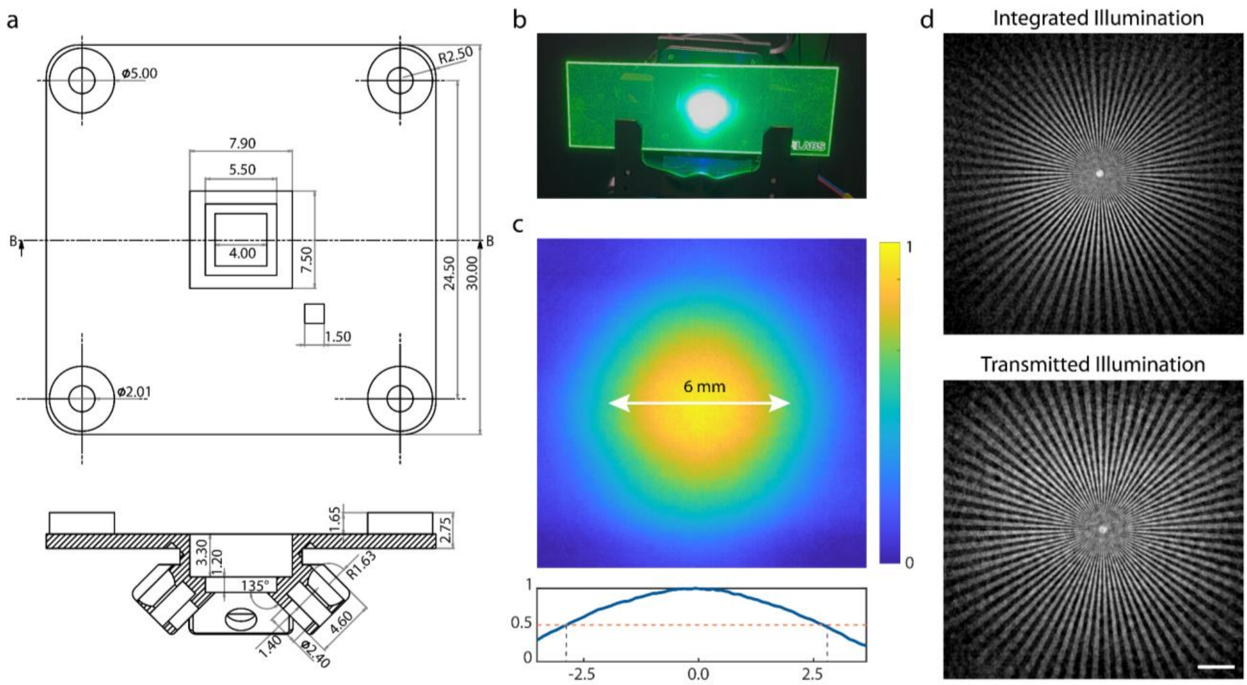

Supplementary Fig. 7 | Bio-FlatScopeNHP housing design and illumination profile. a, Multiview drawing of the 3D printed housing. Unit, mm. **b**, Photo of illumination pattern on a calibration slide. **c**, Experimental capture of illumination pattern by imaging the calibration slide at 3 mm working distance. Unit of the bottom plot, mm. **d**, Reconstructed images of a star target with a 8 mm × 8 mm FOV using integrated illumination and transmitted illumination. It demonstrates that illumination is one of the main factors which limits the current FOV of the system. Scale bar, 1 mm.

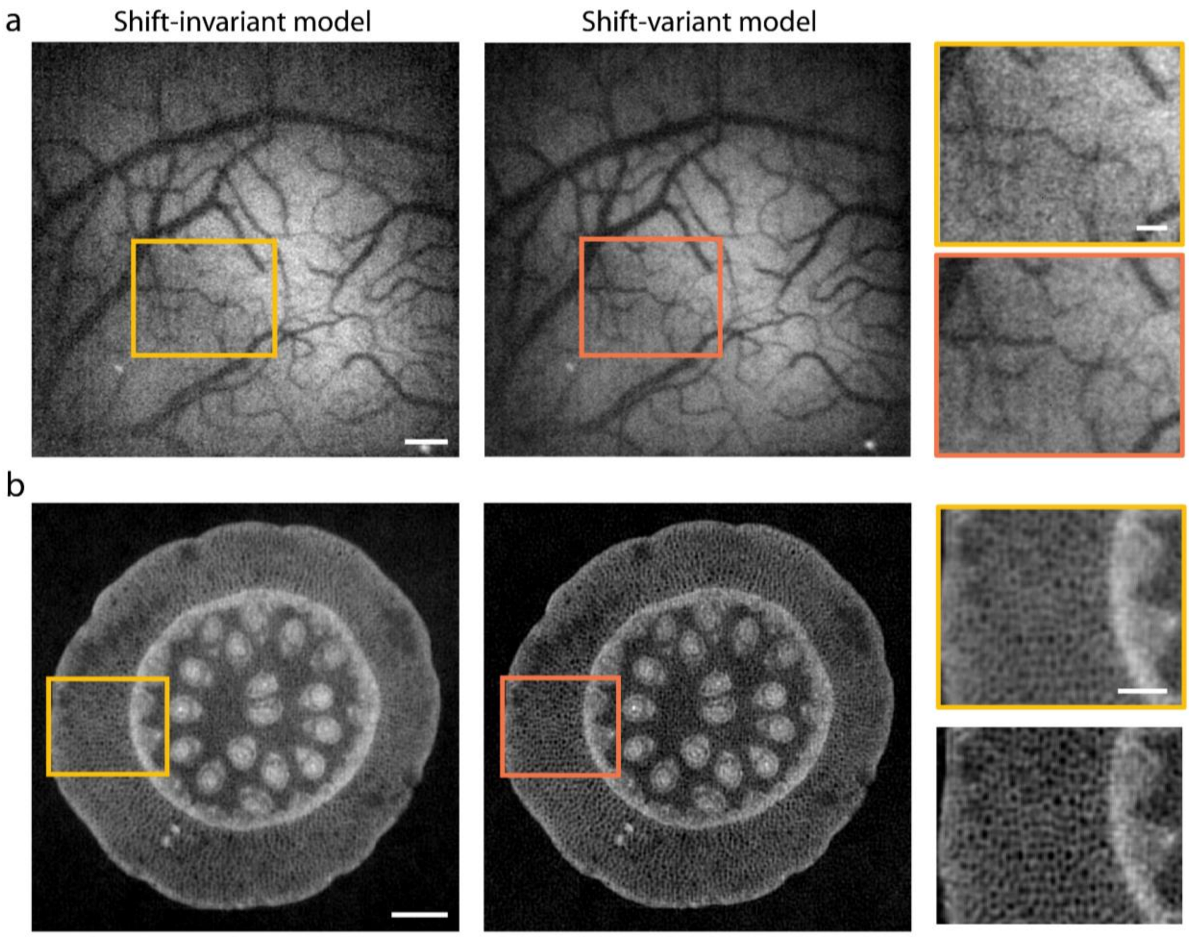

Supplementary Fig. 8 | Comparison between different reconstruction models. a, In vivo fluorescence images captured by Bio-FlatScopeNHP and reconstructed using two different models. Scale bar, 500 μm. Zoom-in scale bar, 200 μm. **b**, A stained slice of Convallaria Rhizome captured by Bio-FlatScopeNHP and reconstructed using two different models. Scale bar, 500 μm. Zoom-in scale bar, 200 μm.

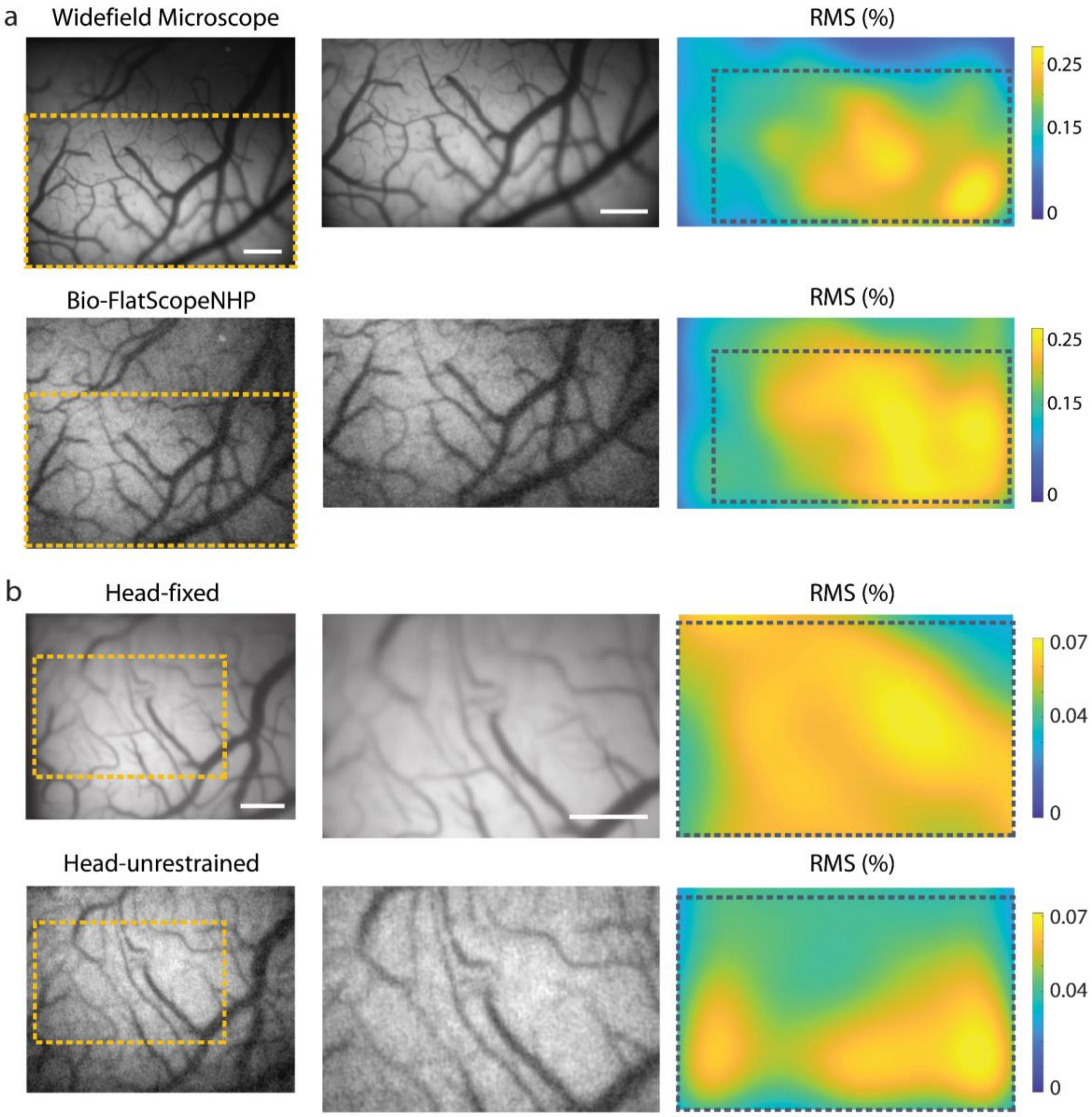

Supplementary Fig. 9 | Root-mean-square (RMS) maps for selecting ROIs. a, Left: Example image captured on a head-fixed macaque by ground truth widefield microscope (top) and Bio-FlatScopeNHP (bottom). Middle: zoom-ins of the yellow square areas. Right: Root mean square (RMS) map used for region of interest (ROI) selection in ground truth widefield microscope (top) and Bio-FlatScopeNHP (bottom). The gray square indicates the selected overlapping ROI. Scale bar, 500 μm. **b**, Left: Example image captured on a head-fixed macaque by ground truth widefield microscope (top) and a head-unrestrained macaque by Bio-FlatScopeNHP (bottom). Middle: zoom-ins of the yellow square areas. Right: Root mean square (RMS) map used for region of interest (ROI) selection in head-fixed session (top) and head-unrestrained session (bottom). The gray square indicates the selected overlapping ROI. Scale bar, 500 μm.

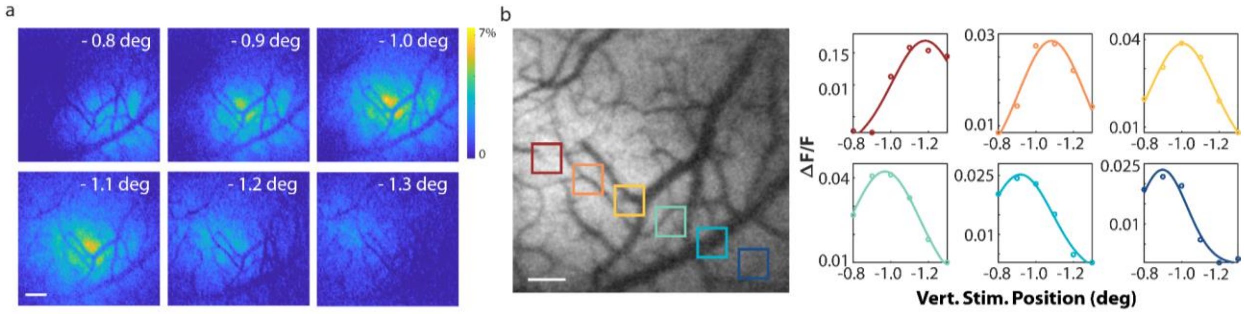

Supplementary Fig. 10 | Additional results for spatial response profiles measured from V1 in the right hemisphere of a head-fixed NHP. a, Response amplitude Bio-FlatScopeNHP at different stimulus conditions in one experiment session. Scale bar, 500 μm. **b,** An example image captured and reconstructed using Bio-FlatScopeNHP (left). Spatial distribution of response amplitudes for different stimulus positions in Bio-FlatScopeNHP reconstructions (right) in one experiment session. Source data are provided as a Source Data file.

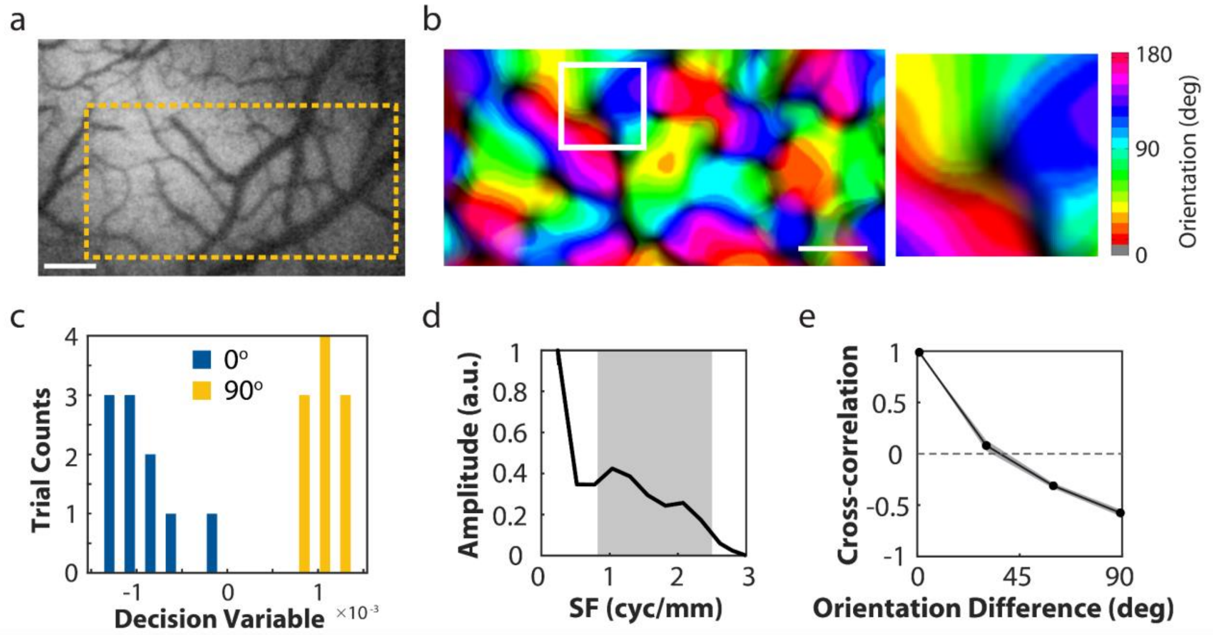

Supplementary Fig. 11 | Additional results for columnar-scale signals measured from V1 in the right hemisphere of a head-fixed NHP. a, Example image captured by Bio-FlatScopeNHP. Scale bar, 500 μm. The yellow square indicates the selected overlapping ROI. **b**, Orientation map obtained by Bio-FlatScopeNHP. Scale bar, 500 μm. **c**, Decision variables calculated from 0-degree and 90-degree trials captured by Bio-FlatScopeNHP. **d**, Normalized amplitude at different spatial frequencies obtained by Bio-FlatScopeNHP. The spatial filtration removes components outside spatial frequency between 0.8 and 2.5 cycles/mm, indicated by the shaded. **e**, Pairwise correlations between all six orientation maps as a function of stimulus orientation difference obtained by Bio-FlatScopeNHP. Shaded area ± SEM. Source data are provided as a Source Data file.

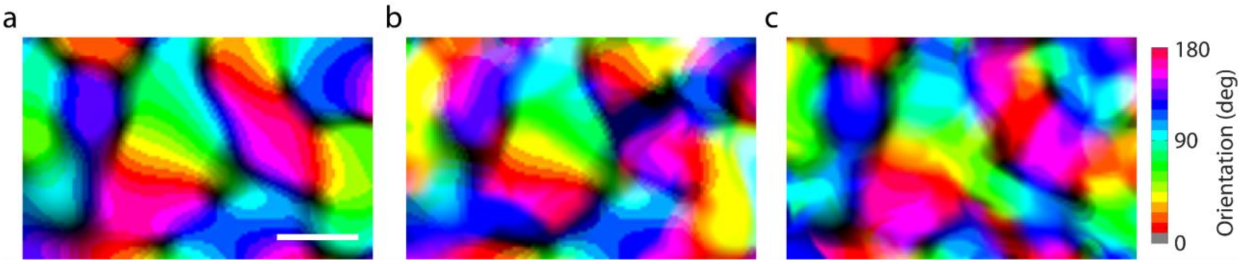

Supplementary Fig. 12 | Comparison between head-fixed and head-unrestrained imaging using Bio-FlatScopeNHP in the same imaging session. a, Orientation map obtained by the ground truth widefield microscope on the head-fixed macaque. Scale bar, 500 µm. **b**, Orientation map obtained by Bio-FlatScopeNHP on the head-fixed macaque. **c**, Orientation map obtained by Bio-FlatScopeNHP on the head-unrestrained macaque.

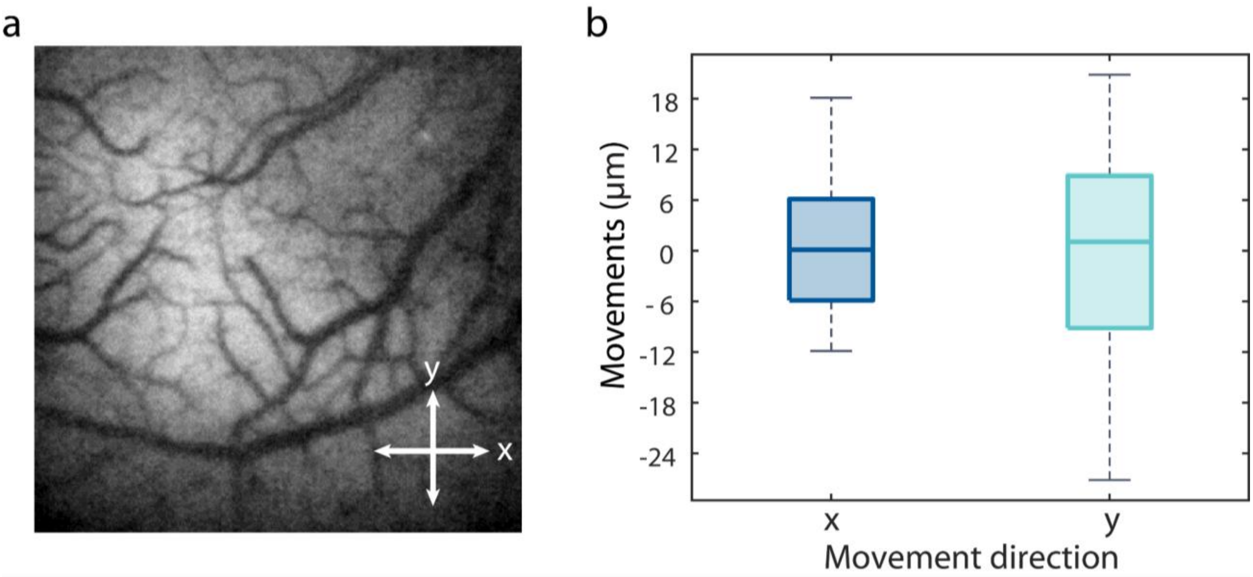

Supplementary Fig. 13 | Stability of Bio-FlatScopeNHP imaging on a head-unrestrained macaque. a, An example reconstruction of the Bio-FlatScope. x direction: medio-latera, y direction: anterior-posterior. **b**, FOV movements in 10 trials (220 frames) in two directions. Source data are provided as a Source Data file.

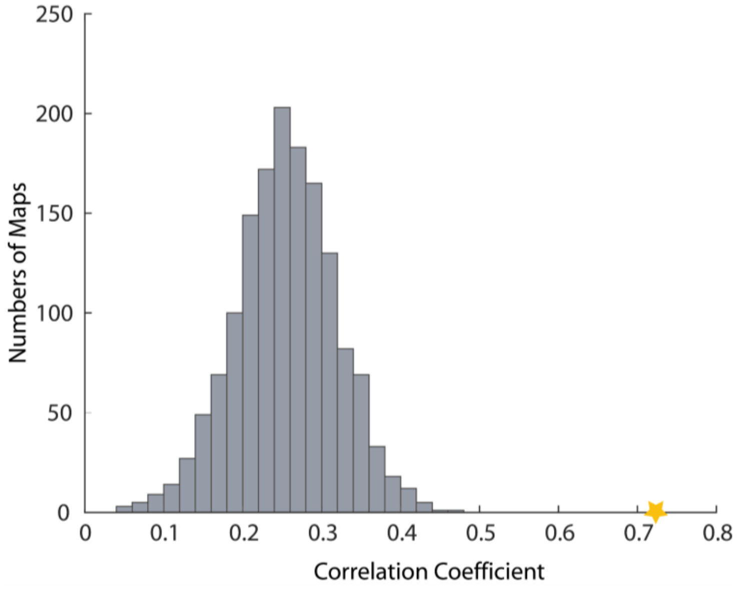

Supplementary Fig. 14 | Correlation coefficient between random maps and ground truth. Yellow star indicates the correlation coefficient obtained from the head-unrestrained macaque by Bio-FlatScopeNHP. Source data are provided as a Source Data file.

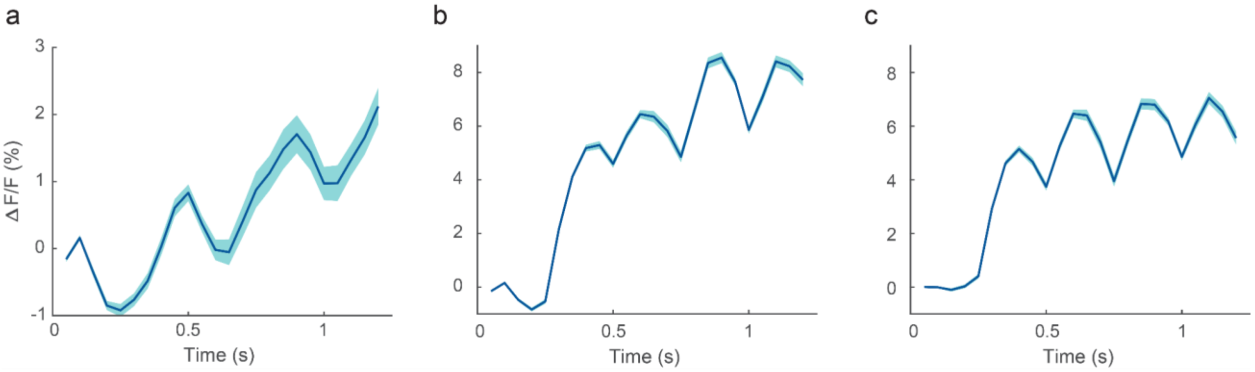

Supplementary Fig. 15 | Heartbeat artifact removal. a, Average time course of blank trials over the center 2 mm × 2 mm area of the FOV. The signal fluctuation is caused by the heartbeat. Shaded area ± SEM. b, Average time course of GCaMP response to the flashed gratings (60 degree stimulus) over the center 2 mm × 2 mm area of the FOV before heartbeat artifact correction. Shaded area ± SEM. c, Average time course of GCaMP response to the flashed gratings (60 degree stimulus) over the center 2 mm × 2 mm area of the FOV after heartbeat artifact correction. Shaded area ± SEM. Data presented here were captured using the table-top widefield microscope. All traces represent average across 10 repeats. Source data are provided as a Source Data file.

Supplementary References:

1. Zhou, Z. C., Yu, C., Sellers, K. K. & Fröhlich, F. Dorso-Lateral Frontal Cortex of the Ferret Encodes Perceptual Difficulty during Visual Discrimination. *Sci. Rep.* 6, 23568 (2016).
2. Scott, B. B. *et al.* Imaging Cortical Dynamics in GCaMP Transgenic Rats with a Head-Mounted Widefield Macroscope. *Neuron* 100, 1045-1058.e5 (2018).
3. Walker, J. D. *et al.* Chronic wireless neural population recordings with common marmosets. *Cell Rep.* 36, 109379 (2021).
4. Gilja, V., Chestek, C. A., Nuyujukian, P., Foster, J. & Shenoy, K. V. Autonomous head-mounted electrophysiology systems for freely behaving primates. *Curr. Opin. Neurobiol.* 20, 676–686 (2010).
5. Kondo, T. *et al.* Calcium Transient Dynamics of Neural Ensembles in the Primary Motor Cortex of Naturally Behaving Monkeys. *Cell Rep.* 24, 2191-2195.e4 (2018).
6. Bollimunta, A. *et al.* Head-mounted microendoscopic calcium imaging in dorsal premotor cortex of behaving rhesus macaque. *Cell Rep.* 35, 109239 (2021).
