# Supplementary material for: Mesoscopic calcium imaging in a head-unrestrained male non-human primate using a lensless microscope
Source: Nat Commun. 2024 Feb 10;15:1271. doi: 10.1038/s41467-024-45417-6 (PMC10858944; doi:10.1038/s41467-024-45417-6)
Supplement: Supplementary file 3 — Reporting Summary [file 41467_2024_45417_MOESM3_ESM.pdf]

nature portfolio

Corresponding author(s): Jacob T. Robinson
Eyal Seidemann

Last updated by author(s): Dec 22, 2023

Reporting Summary

Nature Portfolio wishes to improve the reproducibility of the work that we publish. This form provides structure for consistency and transparency in reporting. For further information on Nature Portfolio policies, see our Editorial Policies and the Editorial Policy Checklist.

Statistics

For all statistical analyses, confirm that the following items are present in the figure legend, table legend, main text, or Methods section.

n/a	Confirmed	
☐	☒	The exact sample size (*n*) for each experimental group/condition, given as a discrete number and unit of measurement
☐	☒	A statement on whether measurements were taken from distinct samples or whether the same sample was measured repeatedly
☒	☐	The statistical test(s) used AND whether they are one- or two-sided<br>*Only common tests should be described solely by name; describe more complex techniques in the Methods section.*
☒	☐	A description of all covariates tested
☐	☒	A description of any assumptions or corrections, such as tests of normality and adjustment for multiple comparisons
☐	☒	A full description of the statistical parameters including central tendency (e.g. means) or other basic estimates (e.g. regression coefficient) AND variation (e.g. standard deviation) or associated estimates of uncertainty (e.g. confidence intervals)
☒	☐	For null hypothesis testing, the test statistic (e.g. *F*, *t*, *r*) with confidence intervals, effect sizes, degrees of freedom and *P* value noted<br>*Give P values as exact values whenever suitable.*
☒	☐	For Bayesian analysis, information on the choice of priors and Markov chain Monte Carlo settings
☒	☐	For hierarchical and complex designs, identification of the appropriate level for tests and full reporting of outcomes
☒	☐	Estimates of effect sizes (e.g. Cohen's *d*, Pearson's *r*), indicating how they were calculated

Our web collection on statistics for biologists contains articles on many of the points above.

Software and code

Policy information about availability of computer code

Data collection	MATLAB, Imaging Source IC Capture, Thorlabs Thorcam
Data analysis	MATLAB. Custom MATLAB codes are available on Github (https://github.com/JiminWu/Bio-FlatScopeNHP)

For manuscripts utilizing custom algorithms or software that are central to the research but not yet described in published literature, software must be made available to editors and reviewers. We strongly encourage code deposition in a community repository (e.g. GitHub). See the Nature Portfolio guidelines for submitting code & software for further information.

Data

Policy information about availability of data

All manuscripts must include a data availability statement. This statement should provide the following information, where applicable:

- Accession codes, unique identifiers, or web links for publicly available datasets
- A description of any restrictions on data availability
- For clinical datasets or third party data, please ensure that the statement adheres to our policy

The main data supporting the results of this study are available within the paper and its Supplementary information. Bio-FlatScopeNHP CAD design, phase mask design, all costume codes and sample data are provided on a Github repository (https://github.com/JiminWu/Bio-FlatScopeNHP). The raw and analyzed datasets generated during the study are too large to be publicly shared, but they are available for research purposes from the corresponding author upon request. Source data are provided with the paper.

Human research participants

Policy information about studies involving human research participants and Sex and Gender in Research.

Reporting on sex and gender	n/a
Population characteristics	n/a
Recruitment	n/a
Ethics oversight	n/a

Note that full information on the approval of the study protocol must also be provided in the manuscript.

Field-specific reporting

Please select the one below that is the best fit for your research. If you are not sure, read the appropriate sections before making your selection.

☒ Life sciences ☐ Behavioural & social sciences ☐ Ecological, evolutionary & environmental sciences

For a reference copy of the document with all sections, see nature.com/documents/nr-reporting-summary-flat.pdf

Life sciences study design

All studies must disclose on these points even when the disclosure is negative.

Sample size	This study focused on the comparison between a new imaging technology and traditional ones, not on the signal comparison between animals. We performed multiple experiments on different days on one animal, which is enough to provide decent signals and comprehensive comparison. The number of sessions is the minimal to compare the technologies. Within each session, the number of trials can provide decent signal to noise ratio (d'>3).
Data exclusions	No data were excluded from the analyses.
Replication	We imaged different regions over a 6 months time period. Results are consistent for all images taken.
Randomization	The animal was picked up based on one traditional imaging technology, by which the clear orientation columns could be observed in at least one GCaMP site (the correlation coefficient is smaller than -0.7 for the orthogonal orientations). The stimulus conditions in each experiment are randomly interleaved.
Blinding	Since the animal is only required to provide decent signals to compare a new imaging technology with traditional ones, the blinding is unnecessary for animal allocation.

Reporting for specific materials, systems and methods

We require information from authors about some types of materials, experimental systems and methods used in many studies. Here, indicate whether each material, system or method listed is relevant to your study. If you are not sure if a list item applies to your research, read the appropriate section before selecting a response.

Materials & experimental systems

n/a	Involved in the study	
☒	☐	Antibodies
☐	☒	Eukaryotic cell lines
☒	☐	Palaeontology and archaeology
☐	☒	Animals and other organisms
☒	☐	Clinical data
☒	☐	Dual use research of concern

Methods

n/a	Involved in the study	
☒	☐	ChIP-seq
☒	☐	Flow cytometry
☒	☐	MRI-based neuroimaging

March 2021

Eukaryotic cell lines

Policy information about cell lines and Sex and Gender in Research

Cell line source(s)	Spiking HEK cells are a gift from Adam Cohen's lab. They are HEK293 cells modified to constitutively express KiR2.1 and NaV1.3 proteins (J. Park et al., PloS ONE, 2013).
Authentication	Cells have been confirmed to be HEK expressing the desired proteins through single cell sequencing
Mycoplasma contamination	All cells are tested negative for mycoplasma contamination.
Commonly misidentified lines (See ICLAC register)	No commonly misidentified lines.

Animals and other research organisms

Policy information about studies involving animals; ARRIVE guidelines recommended for reporting animal research, and Sex and Gender in Research

Laboratory animals	One male rhesus macaque monkey (macaca mulatta, 8 years old) was used for this study
Wild animals	The study did not involve wild animals.
Reporting on sex	One male animal is used for this study.
Field-collected samples	The study did not involve field-collected samples.
Ethics oversight	All Experiments were approved by University of Texas Institutional Animal Care under protocol IAUP-2023-00063 and Use Committee (IACUC) and conform to NIH standards.

Note that full information on the approval of the study protocol must also be provided in the manuscript.
